# Distinct prospective associations of concern over mistakes and doubts about actions with competitive functioning in collegiate athletes: a Bayesian latent-variable study

**DOI:** 10.3389/fpsyg.2026.1961617

**Published:** 2026-10-28

**Authors:** Han Gao, Meijuan Zhang

**Affiliations:** 1 School of Physical Education, Xizang Minzu University, Xianyang, Shaanxi, China; 2 Beijing Union University, Beijing, China

**Keywords:** Bayesian structural equation modeling, choking under pressure, concern over mistakes, doubts about actions, flow, perfectionism, pre-competition anxiety, sport performance

## Abstract

**Background:**

Multidimensional perfectionism theory distinguishes ambitious personal standards from evaluative concerns, but concern over mistakes and doubts about actions may exhibit different prospective associations with competitive functioning. This study tested whether general anxiety symptoms assessed shortly before competition carried distinct indirect associations from these two perfectionism dimensions to post-competition functioning and performance.

**Methods:**

In this three-wave prospective study, 620 collegiate athletes completed item-level measures of personal standards, concern over mistakes, and doubts about actions at baseline. General anxiety symptoms were assessed with the DASS-21 Anxiety subscale 24–48 h before a target competition; flow, study-specific subjective choking, self-rated performance, coach-rated performance, and a harmonized objective performance indicator were assessed within 24 h afterward. A Bayesian ordinal latent-variable partial mediation model adjusted for personal standards, sex, age, competitive level, years in sport, and starter status. Multiple sensitivity analyses evaluated the robustness of the findings.

**Results:**

Concern over mistakes strongly predicted greater anxiety symptoms (beta = 0.609, 95% highest-posterior-density interval [0.472, 0.744]), whereas doubts about actions did not (beta = 0.077, [−0.072, 0.218]). Greater anxiety symptoms predicted lower flow, greater subjective choking, and poorer performance across self-, coach-, and objectively indexed outcomes. Anxiety-related indirect associations from concern over mistakes were supported for flow (beta = −0.225), choking (beta = 0.275), self-rated performance (beta = −0.128), and objective performance (beta = −0.148), but not clearly for coach ratings. No indirect interval for doubts about actions excluded zero, although doubts retained direct associations with all five outcomes. Findings were stable across prior, complete-case, indicator-treatment, external-performance, longer-chain, and bipolar-functioning sensitivity analyses. Flow and subjective choking showed substantial overlap; bifactor results indicated that most of their common variance reflected a general competitive-functioning dimension.

**Conclusion:**

Concern over mistakes demonstrated prospective indirect associations with competitive functioning through general anxiety symptoms assessed before competition and with lower flow, greater subjective choking, and poorer self-rated and objective performance. Mistake-related threat was linked to competitive functioning partly through pre-competition anxiety symptoms, whereas doubts about actions were associated mainly through processes not captured by the general anxiety measure. The findings support treating the two evaluative-concern dimensions as related but psychologically distinct.

## 1 Introduction

Competitive sport requires athletes to pursue exacting standards under conditions where errors are visible, consequential, and often difficult to correct. Perfectionism is therefore especially relevant to sport, but it is not merely a tendency to work hard. Contemporary models distinguish perfectionistic strivings, characterized by demanding personal standards, from perfectionistic concerns, which involve critical reactions to mistakes, perceived discrepancies, and uncertainty about whether actions are correct (Frost et al., 1990; Hewitt and Flett, 1991; Stoeber and Otto, 2006). This distinction matters because high standards can facilitate sustained preparation and goal pursuit, whereas concerns more consistently predict distress and maladjustment (Gotwals et al., 2012; Hill et al., 2018, 2020; Stoeber and Gaudreau, 2017).

Sport-specific evidence reflects this conditional pattern. Perfectionistic strivings have been linked to achievement motivation, deliberate preparation, and sometimes better performance, particularly when maladaptive reactions to imperfection are limited (Gaudreau and Thompson, 2010; Lizmore et al., 2019; Stoeber, 2011; Stoeber et al., 2009). In contrast, perfectionistic concerns have been associated with competitive anxiety, threat-oriented appraisal, burnout, and poorer adjustment after failure (Curran and Hill, 2018; Hill and Curran, 2016; Stoeber et al., 2007). A recent systematic review and meta-analysis found a small positive average association between perfectionistic strivings and sport performance, little overall association for concerns, and substantial heterogeneity across measures and contexts (Kim et al., 2025). Nonlinear results in track-and-field athletes similarly suggest that the impact of high standards on performance depends on their level and evaluative context (Nordin-Bates et al., 2024).

A limitation of the broad strivings-concerns distinction is that it can obscure potentially different components within perfectionistic concerns. Concern over mistakes involves interpreting errors as personally significant failures and anticipating negative consequences from imperfect performance. Doubts about actions refer more directly to uncertainty about whether preparation or execution is adequate. The two constructs are correlated, but they may not be psychologically interchangeable. Concern over mistakes is conceptually close to threat appraisal and worry, whereas doubts about actions may also relate to checking, hesitation, reduced trust in prepared responses, or conscious monitoring of automated skills. When both dimensions are considered simultaneously, their unique coefficients can reveal different residual patterns of association. They cannot, by themselves, determine which psychological processes generated those patterns.

Anxiety is a plausible temporally intermediate correlate, particularly for concerns over mistakes. Attentional control theory proposes that anxiety can shift resources from goal-directed control toward threat monitoring and worry, increasing the effort required to maintain effective task focus (Eysenck et al., 2007). In competitive sport, cognitive anxiety has shown a small negative average association with performance, although its magnitude varies across athletes, tasks, and interpretations of arousal (Craft et al., 2003; Jones, 1995; Woodman and Hardy, 2003). The present study, however, assessed the general Anxiety subscale of the DASS-21 at a pre-competition time point rather than using a sport-specific competitive-anxiety inventory. The mediator is therefore interpreted as general anxiety symptoms assessed 24–48 h before competition, rather than as a direct measure of competition-elicited worry, threat appraisal, or somatic competitive anxiety. Temporal ordering is informative but does not establish a causal mechanism (Maxwell and Cole, 2007).

The downstream outcomes considered here capture psychological experience and performance from several sources. Flow involves deep task absorption, efficient control, and reduced self-conscious monitoring, and is positively associated with performance across sports and other achievement contexts (Harris et al., 2023; Jackson and Eklund, 2002; Norsworthy et al., 2023; Swann et al., 2012). Choking under pressure describes marked deterioration in skilled functioning under pressure and has been theoretically linked to worry, consequences, and explicit control of normally automated actions (Baumeister, 1984; Beilock and Carr, 2001; Hill et al., 2010). In the present study, subjective choking was measured using a study-specific provisional questionnaire, so findings involving this outcome require careful qualification. Performance was represented by athlete self-ratings, coach ratings, and an objective indicator derived from official results. These sources were analyzed separately because they reflect different criteria and methods rather than interchangeable measures of a single performance construct (Schweizer et al., 2020).

Despite a large literature on perfectionism and adjustment, direct tests of temporally ordered indirect pathways in real competitions remain limited. Many studies are cross-sectional, use total or composite scores that overlook item-level measurement error, or rely on a single outcome source. Moreover, analyses frequently combine concern over mistakes and doubts about actions, even though their unique associations may differ. Bayesian latent-variable modeling is useful in this context because it permits simultaneous estimation of ordinal measurement models, correlated outcomes, direct and indirect effects, and posterior uncertainty (Muthén and Asparouhov, 2012; Zyphur and Oswald, 2015). It also encourages transparent inspection of convergence, posterior predictive fit, and prior sensitivity rather than reliance on a single dichotomous test (Depaoli and van de Schoot, 2017).

The present secondary analysis used a three-wave collegiate-athlete cohort centered on a single target competition. Personal standards, concern over mistakes, and doubts about actions were assessed at baseline; general anxiety symptoms were measured 24–48 h before the competition; and flow, subjective choking, and self-, coach-, and objectively indexed performance were assessed within 24 h afterward. We asked four questions. First, did concern over mistakes and doubts about actions uniquely predict general anxiety symptoms assessed before the competition after adjustment for personal standards and pre-specified background variables? Second, were those anxiety symptoms prospectively associated with less favorable post-competition functioning and performance? Third, did anxiety symptoms carry indirect associations from each concern dimension to the five outcomes, and were the indirect effects stronger for concern over mistakes than for doubts about actions? Fourth, did the conclusions remain stable across alternative priors, missing-data restrictions, indicator treatments, external performance outcomes, longer Markov chain Monte Carlo runs, and an alternative representation of flow and choking? We expected concern over mistakes to show a robust positive association with anxiety symptoms and anticipated that anxiety symptoms would predict lower flow, greater choking, and poorer performance. Because theory was less precise about the unique contribution of doubts after controlling for concern over mistakes, the relative CM-DA pattern was treated as a focal research question rather than a preregistered directional hypothesis.

## 2 Method

### 2.1 Participants and procedure

The study used a three-wave prospective observational design centered on one target competition. The baseline sample comprised 620 collegiate athletes aged 17–26 years (*M* = 20.55, SD = 1.69); 360 (58.1%) were men and 260 (41.9%) were women. Competitive level was recorded using the study’s three-category coding: 81 athletes (13.1%) were in a combined master-level/first-level category, 310 (50.0%) were in the second-level category, and 229 (36.9%) were in a combined third-level/general university-team category. Because the sample spanned heterogeneous sports, these categories reflect the study coding rather than a single cross-sport performance standard. Mean formal training experience was 6.45 years (SD = 2.51), and 408 athletes (65.8%) were starters for the target competition. Participant characteristics are summarized in Table 1.

**Table 1:** Sample characteristics, retention, and outcome availability.

Panel A. Sample characteristics				
Characteristic	*n*	*M* or %	SD	Range
Age, years	620	20.55	1.69	17–26
Years in sport	620	6.45	2.51	2–14
Men	360	58.1%	—	—
Women	260	41.9%	—	—
Master-level/first-level category	81	13.1%	—	—
Second-level category	310	50.0%	—	—
Third-level/general university-team category	229	36.9%	—	—
Starter	408	65.8%	—	—
Substitute	212	34.2%	—	—

Panel B. Wave retention and outcome availability		
Stage	*n*	% of baseline
T1: baseline	620	100.0%
T1: complete primary-item wave	554	89.4%
T2: anxiety available	579	93.4%
T2: all anxiety items complete	547	88.2%
T3: any primary outcome	523	84.4%
T3: all primary outcomes complete	464	74.8%

Percentages use the baseline *N* = 620 as the denominator. Competitive-level categories reflect the study coding: category 1 combined master-level/first-level athletes, category 2 included second-level athletes, and category 3 combined third-level/general university-team athletes. T2 and T3 availability indicates at least one relevant variable unless otherwise specified.

Participants were recruited from collegiate athletic programs in northwestern China between September and November 2023. The sample included team sports such as basketball, soccer, and volleyball, as well as individual sports such as track and field, swimming, and racket sports. Eligibility required current participation in collegiate competitive sport, no current sport-related injury, and scheduled participation in the target competition. At Time 1 (T1), athletes completed demographic and multidimensional perfectionism measures. At Time 2 (T2), 24–48 h before the competition, they completed the DASS-21 Anxiety subscale to measure general anxiety symptoms at that time point. At Time 3 (T3), within 24 h after the competition, athletes completed measures of flow, subjective choking, and self-rated performance; coaches provided ratings, and official competition results were recorded. Anxiety data were available for 579 athletes (93.4% of the baseline), and at least one primary T3 outcome was available for 523 athletes (84.4%). The three-wave design and participant retention are summarized in Figure 1.

**Figure 1:**
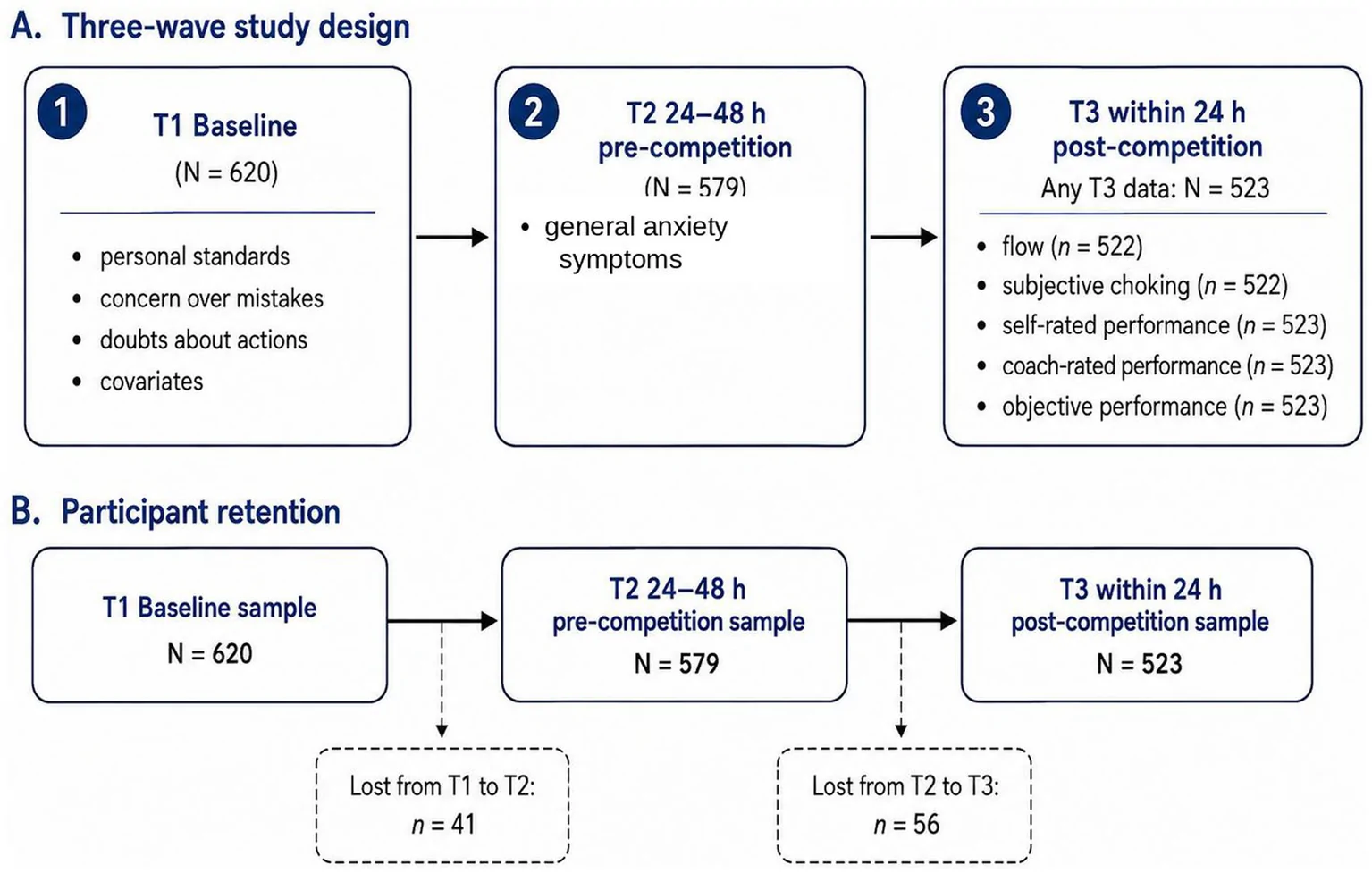
Three-wave design and participant retention. **(A)** Shows the temporal ordering of the focal variables. **(B)** Shows participants with available data. The design is observational, and temporal ordering does not establish causal mediation. Numbers reflect participants with available data at each wave; T3 flow and subjective choking were available for *n* = 522.

The protocol was approved by the Professor Committee of the School of Physical Education, Xizang Minzu University (Approval No. XZMU-IRB-2023-045) and was conducted in accordance with the Declaration of Helsinki. Written informed consent was obtained from adults. Athletes younger than 18 years provided written assent along with written permission from a legal guardian. Since this was a secondary analysis of a fixed cohort, no new prospective power analysis was conducted. Inferential emphasis was placed on posterior precision, convergence, and sensitivity across model specifications.

### 2.2 Measures

#### 2.2.1 Multidimensional perfectionism

Fifteen items drawn from the Sport Multidimensional Perfectionism Scale-2 framework assessed personal standards, concern over mistakes, and doubts about actions, with five items each (Dunn et al., 2006; Gotwals and Dunn, 2009). Responses ranged from 1 to 5. One personal-standards item was reverse scored. Higher scores indicated greater levels of the respective construct. Coefficient alpha/omega were 0.865/0.867 for personal standards, 0.877/0.878 for concern over mistakes, and 0.861/0.860 for doubts about actions (Dunn et al., 2014).

#### 2.2.2 General anxiety symptoms assessed before competition

The seven-item Anxiety subscale of the Depression Anxiety Stress Scales-21 was administered at T2, 24–48 h before the target competition (DASS-21; Lovibond and Lovibond, 1995a,b). Items were rated from 0 to 3. The DASS-21 Anxiety subscale was used to assess general anxiety symptoms at this pre-competition time point. Previous research supports the construct validity of the DASS-21 in nonclinical populations (Henry and Crawford, 2005). Alpha/omega were 0.763/0.762.

#### 2.2.3 Post-competition psychological functioning

State flow was assessed with a nine-item adaptation of the Psychological Flow Scale (Norsworthy et al., 2023), covering absorption, effortless control, and intrinsic reward. Responses used a 1–5 format, with alpha/omega coefficients of 0.847/0.847. The measure was translated into Chinese using forward and backward translation. Subjective choking was assessed with a five-item, study-specific Competition Subjective Choking Questionnaire rated from 1 to 5. The items were developed to capture the perceived experience of performing below one’s capability under pressure, informed by explicit-monitoring accounts of choking (Baumeister, 1984), and reviewed by three sport-psychology experts before use. Alpha/omega coefficients were 0.760/0.761. The measure demonstrated acceptable preliminary internal consistency, factor loadings, and composite reliability. Further independent validation is discussed in the limitations. The complete item set is available from the corresponding author upon reasonable request.

#### 2.2.4 Competition performance

Self-rated and coach-rated performance were study-specific single-item indicators rather than adaptations of previously validated multi-item performance scales. Athletes rated their performance as a percentage of their usual training level on a 0–110% digital slider; values above 100% allowed for performance exceeding the athlete’s typical training baseline. Coaches rated each athlete’s overall performance from 1 to 10. These ratings were modeled separately as complementary athlete and coach perspectives (Schweizer et al., 2020). The objective performance variable was a precomputed standardized score derived from official competition results. Scores were standardized within sport/event/sex groups, with direction harmonized so that higher values represented better performance; for example, lower race times were reversed in direction before standardization. The objective indicator is therefore used as a harmonized cross-sport criterion rather than a raw performance metric.

#### 2.2.5 Covariates

Pre-specified covariates included sex, centered age, competitive level, centered years in sport, and starter status. Sex was coded as female versus male; competitive level was represented by indicators for the second-level category and the third-level/general university-team category, with the combined master-class/first-level category as the reference; and substitute status was contrasted with starter status.

### 2.3 Data preparation and missingness

All variables were checked against their permissible response ranges, and the personal-standards reverse item was recoded before descriptive scale scores were formed. Descriptive means were calculated when at least four of five perfectionism items, six of seven anxiety items, seven of nine flow items, or four of five choking items were available. The primary latent-variable model used item-level data and the Mplus missing-data machinery under the model’s missing-at-random assumptions. Complete item-level cases were reserved for sensitivity analysis rather than being used as the primary sample.

Missingness was evaluated using logistic regressions with continuous predictors standardized. Greater baseline concern over mistakes predicted missing T2 anxiety data (OR = 1.61 per SD, 95% CI [1.05, 2.45]). Among T2 respondents, greater anxiety predicted T3 missingness (OR = 1.68, 95% CI [1.20, 2.35]), as did years in sport (OR = 1.46, 95% CI [1.08, 1.96]). These results argue against a missing-completely-at-random interpretation. The primary analyses therefore rely on a missing-at-random assumption conditional on the variables included in the model; the complete-item-case model assesses sensitivity to a stricter data restriction.

### 2.4 Statistical analysis

Analyses were conducted in Mplus 8.3 (Muthén and Muthén, 2017) and Python. Descriptive reliability estimates, correlations, attrition models, variance-inflation factors, and item-level discriminant-validity diagnostics were generated from the scored dataset. The primary measurement model specified six correlated latent variables: personal standards, concern over mistakes, doubts about actions, general anxiety symptoms assessed before competition, state flow, and subjective choking. Questionnaire items were treated as ordered categorical and estimated using a probit link. The three performance indicators were considered observed continuous outcomes.

The primary structural model was a Bayesian latent partial-mediation model. General anxiety symptoms were regressed on concern over mistakes, doubts about actions, personal standards, and the covariates. Each of the five T3 outcomes was regressed on anxiety symptoms, the three perfectionism factors, and the covariates. The three perfectionism factors were allowed to covary. Residual covariances were estimated among all T3 outcomes because they were assessed after the same competition and represented overlapping but non-identical criteria. For each outcome, the model estimated the indirect effects of concern over mistakes and doubts about actions through anxiety symptoms, along with the corresponding direct effects, total effects, and the direct contrast between the two unstandardized indirect effects (Miočević et al., 2018). These structural paths are interpreted as prospective statistical associations rather than identified psychological mechanisms.

The primary Bayesian model used Mplus default priors, four Markov chains, a minimum of 10,000 iterations, thinning by 10, and posterior medians as point estimates. A fixed 20,000-iteration replication was subsequently run to verify stability. Convergence was evaluated from TECH8 using the potential scale reduction factor (PSR), with values near 1.00 and below approximately 1.01 preferred for the final runs. Model assessment used the posterior predictive *p*-value (PPP) and the 95% interval for the difference between observed and replicated Chi-square values. Standardized STDYX posterior medians, posterior standard deviations, and 95% highest-posterior-density (HPD) intervals are reported. An effect was considered supported when its 95% HPD interval excluded zero; Mplus one-tailed posterior *p*-values were not used as the primary decision criterion. Bayesian reporting followed the transparency principles described by Depaoli and van de Schoot (2017).

Sensitivity analyses included a model fixing direct perfectionism-to-outcome paths to zero, a direct-only model omitting anxiety as a predictor of T3 outcomes, normal *N* (0, 1) priors on focal structural paths, complete item-level cases (*n* = 391), a model limited to coach-rated and objective performance, and a model treating indicators as continuous. Measurement sensitivity compared the six-factor model with a model combining flow and reverse-oriented choking items into a bipolar competitive-functioning factor using WLSMV and the Mplus DIFFTEST procedure. A bifactor model with a general competitive-functioning factor plus flow- and choking-specific factors was also estimated. Finally, the structural model was re-estimated with the bipolar functioning factor and 20,000 fixed iterations.

Multicollinearity among the three perfectionism scale scores was modest (VIFs = 1.27–1.68). Approximate composite reliability and average variance extracted were calculated from standardized loadings. Fornell-Larcker and item-level HTMT diagnostics were used as descriptive complements to the ordinal latent models (Fornell and Larcker, 1981; Henseler et al., 2015). In the bifactor sensitivity model, explained common variance was calculated as the proportion of squared common-factor loadings attributable to the general factor (Rodriguez et al., 2016).

### 2.5 Transparency and reproducibility

The study and the present secondary analysis were not preregistered. Analysis code, Mplus inputs and outputs, automated checks, and manuscript tables were retained in a versioned archive and are available from the corresponding author upon reasonable request. Participant-level data are not publicly posted because the records contain potentially identifying information about collegiate athletes; de-identified access is subject to institutional and confidentiality restrictions. The objective performance variable in the analysis archive is the precomputed standardized score.

## 3 Results

### 3.1 Preliminary analyses and measurement quality

Sample characteristics and retention are shown in Table 1 and Figure 1. At least one anxiety observation was retained for 93.4% of the baseline sample, and at least one T3 outcome was retained for 84.4%. Descriptive statistics, reliability estimates, and pairwise correlations appear in Table 2. Concern over mistakes and doubts about actions were strongly correlated (*r* = 0.61) and showed the most consistent bivariate associations with subsequent outcomes. Both were associated with increased anxiety and choking, as well as decreased flow and poorer performance. Personal standards correlated weakly with anxiety (*r* = 0.15) and choking (*r* = 0.17) but not materially with flow or performance. Flow correlated −0.76 with subjective choking and 0.55–0.59 with the three performance indicators.

**Table 2:** Descriptive statistics, internal consistency, and correlations.

Variable	*M*	SD	Alpha	1	2	3	4	5	6	7	8	9
				PS	CM	DA	ANX	FLOW	CHOKE	SELF	COACH	OBJ
1. Personal standards	3.62	0.85	0.865	—	—	—	—	—	—	—	—	—
2. Concern over mistakes	2.84	0.93	0.877	0.418	—	—	—	—	—	—	—	—
3. Doubts about actions	2.70	0.87	0.861	0.405	0.608	—	—	—	—	—	—	—
4. Anxiety symptoms (T2)	1.19	0.54	0.763	0.153	0.495	0.354	—	—	—	—	—	—
5. State flow	3.29	0.62	0.847	0.026	−0.439	−0.428	−0.468	—	—	—	—	—
6. Subjective choking	2.83	0.68	0.760	0.166	0.470	0.471	0.494	−0.763	—	—	—	—
7. Self-rated performance	81.68	9.94	—	0.049	−0.272	−0.295	−0.296	0.575	−0.495	—	—	—
8. Coach-rated performance	6.78	1.23	—	0.030	−0.320	−0.293	−0.282	0.592	−0.516	0.415	—	—
9. Objective performance	0.07	0.74	—	0.021	−0.312	−0.280	−0.332	0.547	−0.508	0.369	0.430	—

Pairwise *n* ranged from 521 to 619. Objective performance is a precomputed standardized score that was standardized within sport/event/sex groups. Internal consistency is not applicable to the single-item performance indicators.

The Bayesian six-factor measurement model terminated normally (PPP = 0.632; observed-minus-replicated discrepancy interval [−115.88, 82.08]; final PSR = 1.003). Standardized loadings ranged from 0.745 to 0.830 for personal standards, 0.782 to 0.824 for concern over mistakes, 0.726 to 0.783 for doubts about actions, 0.562 to 0.628 for anxiety symptoms, 0.622 to 0.694 for flow, and 0.624 to 0.706 for choking. Approximate composite reliability ranged from 0.79 to 0.90. Average variance extracted was above 0.50 for the three perfectionism factors but below 0.50 for anxiety symptoms, flow, and choking. For the study-specific choking measure, these indices constitute preliminary within-sample internal structure evidence only; they do not substitute for independent construct validation.

The principal measurement concern was the overlap between flow and subjective choking. Their Bayesian latent correlation was −0.908, 95% HPD [−0.936, −0.876], and the item-level HTMT approximation was 0.952. A WLSMV six-factor model fit well, Chi-square (579) = 538.92, *p* = 0.882, CFI = 1.000, TLI = 1.003, RMSEA = 0.000, 90% CI [0.000, 0.007], SRMR = 0.028, but the flow-choking latent correlation was −0.954. A bipolar functioning model also showed good absolute fit, Chi-square (584) = 645.80, *p* = 0.039, CFI = 0.996, TLI = 0.995, RMSEA = 0.013, 90% CI [0.003, 0.019], SRMR = 0.034. WLSMV DIFFTEST favored the less restricted six-factor model, delta Chi-square (5) = 60.87, *p* < 0.001. A bifactor model fit well, Chi-square (63) = 59.63, *p* = 0.597, CFI = 1.000, TLI = 1.001, RMSEA = 0.000, 90% CI [0.000, 0.024], SRMR = 0.018; general-factor loadings ranged in absolute magnitude from 0.594 to 0.707, and the approximate explained common variance was 0.923. Thus, statistically detectable specific variance remained, but most common flow-choking variance reflected a bipolar competitive-functioning dimension. Flow and choking are therefore not treated as two fully independent replications of the same structural pattern (Table 3 and Figure 2).

**Table 3:** Measurement quality and model diagnostics.

Panel A. Bayesian measurement quality.				
Factor	Items	Loading range	Composite reliability	AVE
Personal standards	5	0.745–0.830	0.888	0.613
Concern over mistakes	5	0.782–0.824	0.896	0.634
Doubts about actions	5	0.726–0.783	0.877	0.587
Anxiety symptoms (T2)	7	0.562–0.628	0.793	0.354
State flow	9	0.622–0.694	0.871	0.428
Subjective choking	5	0.624–0.706	0.801	0.447

Panel B. Measurement and structural model diagnostics.			
Model	*N*	Fit/Predictive check	Additional diagnostic
Bayesian six-factor measurement	620	PPP = 0.632; discrepancy [−115.88, 82.08]	PSR = 1.003
Bayesian primary structural	620	PPP = 0.665; discrepancy [−144.80, 97.75]	PSR = 1.006; 20 k PSR = 1.002
WLSMV six-factor measurement	620	Chi-square (579) = 538.92, *p* = 0.882; CFI = 1.000; TLI = 1.003	RMSEA = 0.000 [0.000,0.007]; SRMR = 0.028
WLSMV bipolar functioning	620	Chi-square (584) = 645.80, *p* = 0.039; CFI = 0.996; TLI = 0.995	RMSEA = 0.013 [0.003,0.019]; SRMR = 0.034
WLSMV DIFFTEST	620	Delta Chi-square (5) = 60.87, *p* < 0.001	Six-factor favored
WLSMV bifactor functioning	523	Chi-square (63) = 59.63, *p* = 0.597; CFI = 1.000; TLI = 1.001	RMSEA = 0.000 [0.000,0.024]; SRMR = 0.018; ECV = 0.923

AVE, average variance extracted; ECV, explained common variance; PPP, posterior predictive *p*-value; PSR, potential scale reduction factor; WLSMV, weighted least squares mean- and variance-adjusted. ANX is the DASS-21 general anxiety-symptom factor measured 24–48 h before competition. Bayesian flow-choking latent *r* = −0.908, 95% HPD [−0.936, −0.876]; WLSMV *r* = −0.954; item-level HTMT approximation = 0.952.

**Figure 2:**
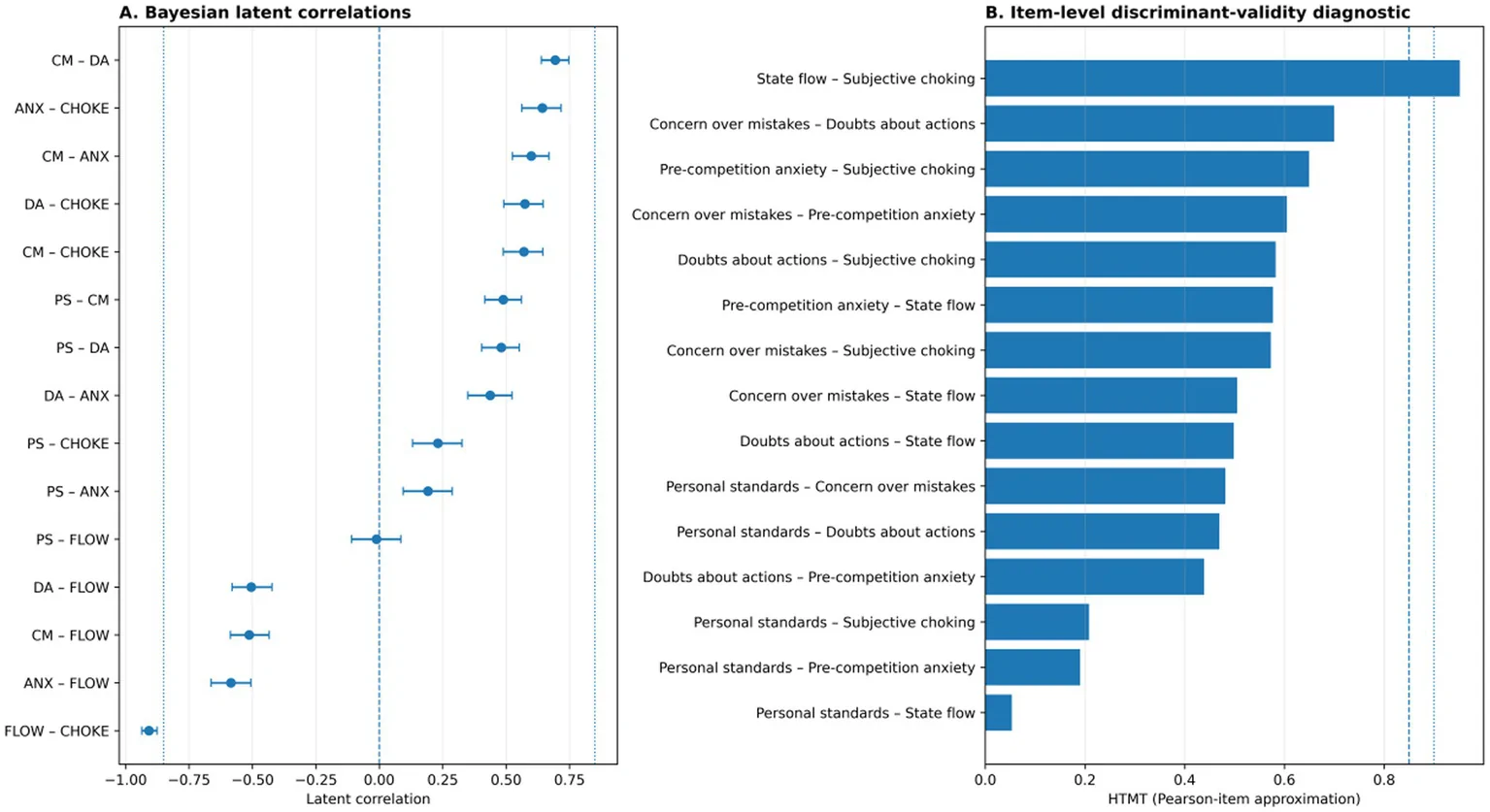
Measurement and discriminant-validity diagnostics. **(A)** Shows selected Bayesian latent correlations and 95% HPD intervals. **(B)** Presents approximate item-level HTMT values. The flow-choking pair exceeded conventional discriminant-validity guidelines; alternative WLSMV and bifactor results are reported in Table 3.

### 3.2 Primary structural paths

The primary partial-mediation model terminated normally. PPP was 0.665, and the 95% posterior predictive discrepancy interval [−144.80, 97.75] included zero. The final PSR was 1.006 after the initial run and 1.002 in the fixed 20,000-iteration replication, indicating stable chain mixing. Standardized focal paths are reported in Table 4 and Figure 3.

**Table 4:** Standardized structural paths and explained variance in the primary model.

Outcome	Predictor	Beta	Posterior SD	95% HPD	Interpretation
Anxiety symptoms	Concern over mistakes	0.609	0.069	[0.472, 0.744]	Supported
Anxiety symptoms	Doubts about actions	0.077	0.074	[−0.072, 0.218]	Not supported
Anxiety symptoms	Personal standards	−0.146	0.055	[−0.255, −0.038]	Supported
State flow	Anxiety symptoms	−0.374	0.061	[−0.491, −0.257]	Supported
State flow	Concern over mistakes	−0.191	0.081	[−0.350, −0.032]	Supported
State flow	Doubts about actions	−0.370	0.065	[−0.493, −0.239]	Supported
State flow	Personal standards	0.339	0.051	[0.240, 0.439]	Supported
Subjective choking	Anxiety symptoms	0.456	0.063	[0.331, 0.577]	Supported
Subjective choking	Concern over mistakes	0.083	0.086	[−0.091, 0.250]	Not supported
Subjective choking	Doubts about actions	0.345	0.069	[0.209, 0.478]	Supported
Subjective choking	Personal standards	−0.067	0.055	[−0.175, 0.039]	Not supported
Self-rated performance	Anxiety symptoms	−0.213	0.065	[−0.336, −0.080]	Supported
Self-rated performance	Concern over mistakes	−0.084	0.085	[−0.254, 0.080]	Not supported
Self-rated performance	Doubts about actions	−0.289	0.069	[−0.421, −0.153]	Supported
Self-rated performance	Personal standards	0.253	0.054	[0.146, 0.357]	Supported
Coach-rated performance	Anxiety symptoms	−0.131	0.065	[−0.258, −0.004]	Supported
Coach-rated performance	Concern over mistakes	−0.242	0.083	[−0.407, −0.082]	Supported
Coach-rated performance	Doubts about actions	−0.219	0.068	[−0.353, −0.084]	Supported
Coach-rated performance	Personal standards	0.259	0.053	[0.155, 0.361]	Supported
Objective performance	Anxiety symptoms	−0.246	0.065	[−0.371, −0.117]	Supported
Objective performance	Concern over mistakes	−0.162	0.084	[−0.319, 0.012]	Not supported
Objective performance	Doubts about actions	−0.187	0.069	[−0.319, −0.049]	Supported
Objective performance	Personal standards	0.213	0.053	[0.110, 0.317]	Supported

Explained variance		
Outcome	*R*-squared	95% HPD
General anxiety symptoms (T2)	0.384	[0.295, 0.467]
State flow	0.526	[0.443, 0.606]
Subjective choking	0.540	[0.452, 0.626]
Self-rated performance	0.221	[0.152, 0.290]
Coach-rated performance	0.226	[0.160, 0.297]
Objective performance	0.222	[0.155, 0.290]

STDYX posterior medians are shown. Models adjusted for sex, centered age, competitive level, centered years in sport, and substitute status. Interpretation is based on whether the 95% HPD interval excludes zero.

**Figure 3:**
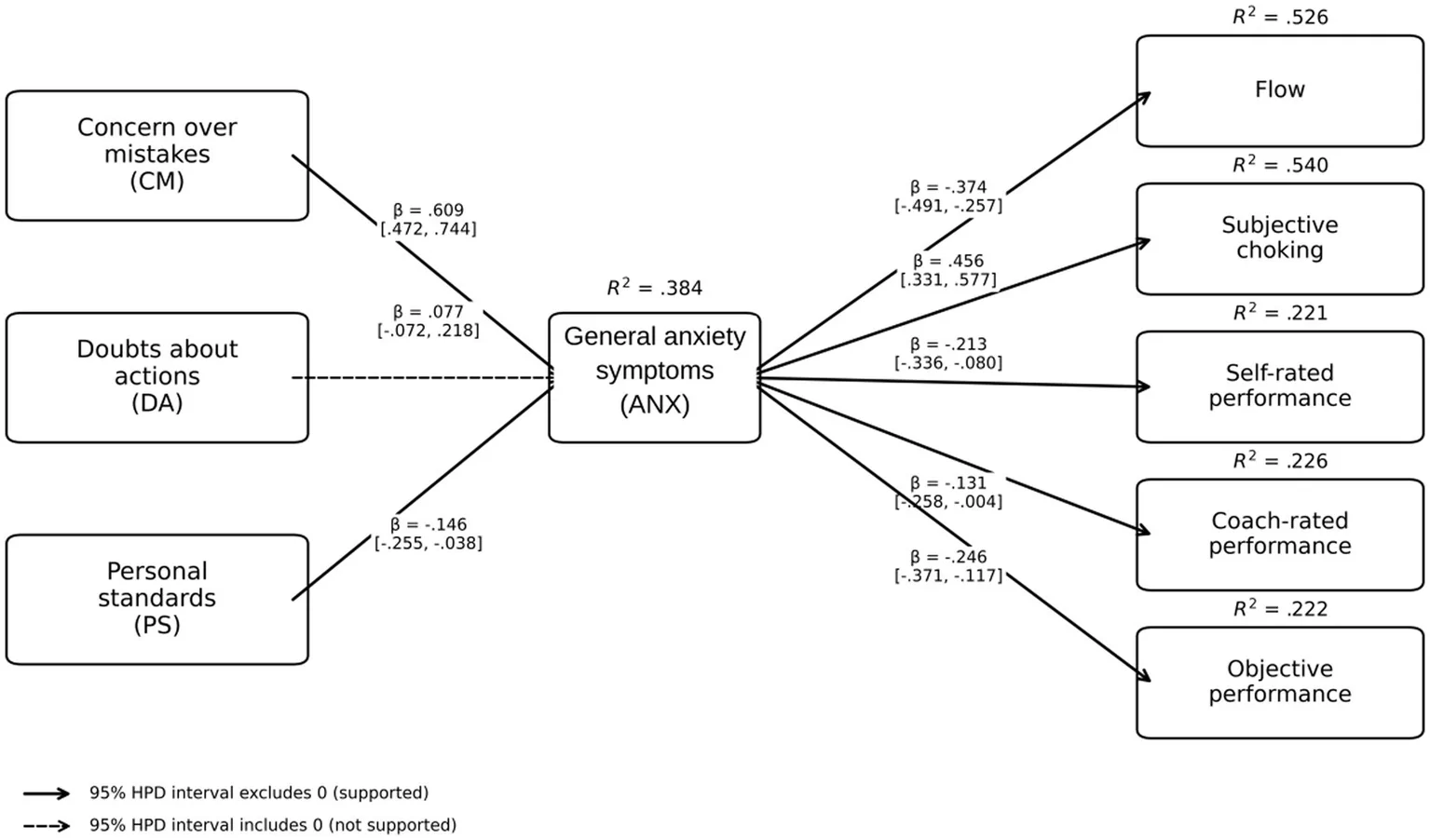
Standardized primary Bayesian latent mediation model. Values are standardized STDYX posterior medians with 95% HPD intervals. ANX denotes general anxiety symptoms measured with the DASS-21 24–48 h before competition. Solid paths have intervals excluding zero; the dashed DA-to-ANX path includes zero. Direct perfectionism-to-outcome paths, covariates, and residual covariances were estimated but omitted for clarity.

Concern over mistakes strongly predicted greater general anxiety symptoms assessed before competition (beta = 0.609, 95% HPD [0.472, 0.744]). Doubts about actions did not maintain a supported unique path after controlling for concern over mistakes and personal standards (beta = 0.077, 95% HPD [−0.072, 0.218]). Personal standards predicted lower anxiety symptoms after the concern dimensions were controlled (beta = −0.146, 95% HPD [−0.255, −0.038]). The model explained 38.4% of the variance in anxiety symptoms.

Greater anxiety symptoms predicted less favorable functioning on every T3 criterion: lower flow (beta = −0.374, 95% HPD [−0.491, −0.257]), greater subjective choking (beta = 0.456, [0.331, 0.577]), poorer self-rated performance (beta = −0.213, [−0.336, −0.080]), poorer coach-rated performance (beta = −0.131, [−0.258, −0.004]), and poorer objective performance (beta = −0.246, [−0.371, −0.117]). The model explained 52.6% of flow, 54.0% of choking, and approximately 22% of each performance indicator.

Direct paths revealed further differentiation. Concern over mistakes retained direct associations with lower flow (beta = −0.191) and poorer coach ratings (beta = −0.242), whereas its direct intervals included zero for choking, self-rated performance, and objective performance. Doubts about actions maintained direct associations with all five outcomes: less flow (beta = −0.370), greater choking (beta = 0.345), and poorer self-rated (beta = −0.289), coach-rated (beta = −0.219), and objective performance (beta = −0.187). Personal standards predicted more flow and better performance across all three sources after controlling for the concern dimensions; its direct path to choking was not supported.

### 3.3 Prospective indirect effects

Table 5 and Figure 4 report the standardized indirect effects. Concern over mistakes showed indirect associations that were supported through general anxiety symptoms assessed before competition, leading to lower flow (beta = −0.225, 95% HPD [−0.324, −0.140]), greater choking (beta = 0.275, [0.177, 0.384]), poorer self-rated performance (beta = −0.128, [−0.218, −0.046]), and poorer objective performance (beta = −0.148, [−0.237, −0.066]). The indirect effect for coach-rated performance was borderline but did not meet the pre-specified interval criterion (beta = −0.078, 95% HPD [−0.160, 0.001]).

**Table 5:** Standardized indirect, direct, and total effects.

Predictor	Outcome	Indirect beta	Indirect 95% HPD	Direct beta	Direct 95% HPD	Total beta	Total 95% HPD	CM-DA indirect contrast
Concern over mistakes	State flow	−0.225	[−0.324, −0.140]	−0.191	[−0.350, −0.032]	−0.418	[−0.548, −0.283]	−0.140 [−0.245, −0.045]
Concern over mistakes	Subjective choking	0.275	[0.177, 0.384]	0.083	[−0.091, 0.250]	0.361	[0.214, 0.500]	0.162 [0.058, 0.284]
Concern over mistakes	Self-rated performance	−0.128	[−0.218, −0.046]	−0.084	[−0.254, 0.080]	−0.213	[−0.348, −0.071]	−0.086 [−0.176, −0.020]
Concern over mistakes	Coach-rated performance	−0.078	[−0.160, 0.001]	−0.242	[−0.407, −0.082]	−0.321	[−0.455, −0.187]	−0.052 [−0.125, 0.003]
Concern over mistakes	Objective performance	−0.148	[−0.237, −0.066]	−0.162	[−0.319, 0.012]	−0.311	[−0.448, −0.176]	−0.101 [−0.192, −0.027]
Doubts about actions	State flow	−0.028	[−0.083, 0.028]	−0.370	[−0.493, −0.239]	−0.398	[−0.528, −0.267]	—
Doubts about actions	Subjective choking	0.034	[−0.033, 0.102]	0.345	[0.209, 0.478]	0.379	[0.242, 0.522]	—
Doubts about actions	Self-rated performance	−0.015	[−0.052, 0.016]	−0.289	[−0.421, −0.153]	−0.305	[−0.441, −0.169]	—
Doubts about actions	Coach-rated performance	−0.008	[−0.036, 0.011]	−0.219	[−0.353, −0.084]	−0.229	[−0.364, −0.094]	—
Doubts about actions	Objective performance	−0.018	[−0.059, 0.018]	−0.187	[−0.319, −0.049]	−0.206	[−0.337, −0.063]	—

The CM-DA contrast is an unstandardized MODEL CONSTRAINT difference and is displayed only on the concern-over-mistakes row for each outcome. Four of five contrast intervals excluded zero. The CM coach-rating indirect interval included zero and was treated as unsupported.

**Figure 4:**
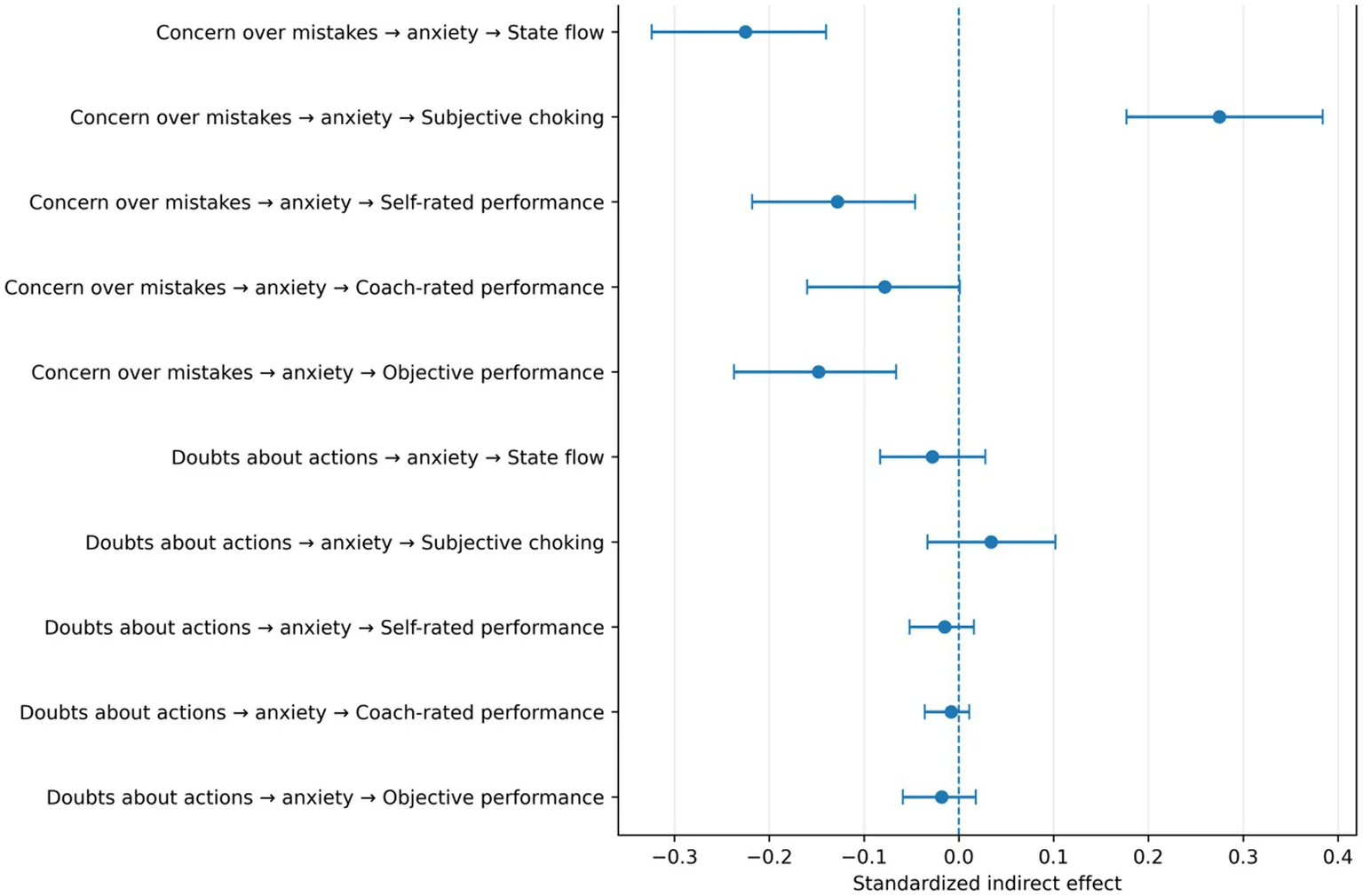
Standardized indirect effects through general anxiety symptoms assessed before competition. Points are standardized indirect posterior medians; bars are 95% HPD intervals. The mediator is the DASS-21 general anxiety-symptom factor measured 24–48 h before competition. The vertical dashed line denotes zero.

None of the five doubts-about-actions indirect intervals through anxiety symptoms excluded zero. Estimates ranged from beta = −0.028 for flow to beta = 0.034 for choking, and all intervals crossed zero. Direct unstandardized contrasts of the CM and DA indirect effects supported differences for flow, choking, self-rated performance, and objective performance; the contrast for coach-rated performance included zero. These contrasts establish different modeled indirect associations for four outcomes but do not identify the psychological processes responsible for the remaining direct DA associations.

### 3.4 Robustness and additional analyses

The core concern-over-mistakes indirect effects through anxiety symptoms were highly stable. The paths to flow, choking, self-rated performance, and objective performance were supported in the regularizing-prior, complete-item-case, and continuous-indicator models; the objective performance effect was also supported in the external-performance model. The coach-rating indirect effect was supported in only one of five specifications and was therefore treated as inconclusive. No doubts-about-actions indirect effect was supported in any comparable sensitivity model (Figure 5).

**Figure 5:**
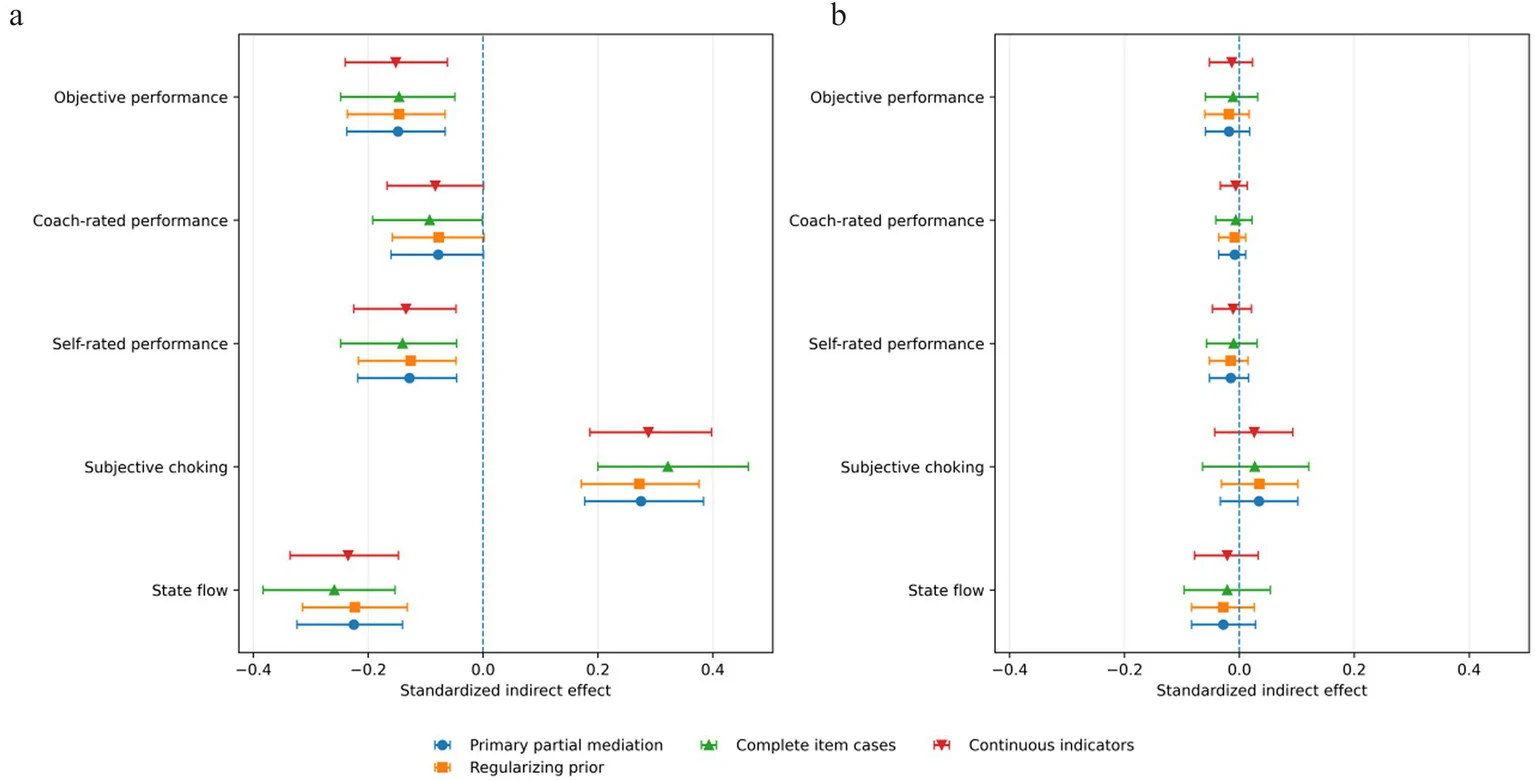
Robustness of indirect effects across model specifications. Points and intervals compare the primary, regularizing-prior, complete-item-case, and continuous-indicator models. All indirect effects use the DASS-21 general anxiety-symptom factor as the mediator. The external-performance-only model is summarized in Table 5 because it estimated only coach-rated and objective performance. **(a)** Concern over mistakes. **(b)** Doubts about actions.

The forced full-mediation model produced supported DA indirect effects only after all direct perfectionism-to-outcome paths were constrained to zero. Because the primary model retained substantial direct DA paths, this result is best regarded as a consequence of the full-mediation constraint rather than evidence of a stable DA indirect pathway. The 20,000-iteration replication reproduced the primary model’s path estimates and fit. In the bipolar structural sensitivity model, PPP was 0.496 and final PSR was 1.006. Anxiety symptoms predicted lower overall competitive functioning (beta = −0.409, 95% HPD [−0.521, −0.298]); the CM indirect effect remained supported (beta = −0.246, [−0.343, −0.160]), whereas the DA indirect effect did not (beta = −0.031, [−0.094, 0.027]). Thus, the differentiated CM-DA statistical pattern was retained when flow and choking were represented by a single bipolar functioning factor.

## 4 Discussion

### 4.1 Principal findings and differentiated pathways

The present study examined whether two components of perfectionistic concerns exhibited the same prospective statistical pattern in relation to competitive functioning. The results revealed different patterns. Concern over mistakes strongly predicted greater general anxiety symptoms assessed shortly before competition, and those symptoms were associated with less flow, greater subjective choking, and poorer performance across self-, coach-, and objective indicators. Indirect associations from concern over mistakes through anxiety symptoms were supported to four of the five outcomes. In contrast, doubts about actions did not uniquely predict anxiety symptoms and showed no supported anxiety-related indirect effect, yet retained direct associations with every outcome. Direct contrasts confirmed that the indirect effects of CM and DA differed for four outcomes. The central result is therefore a difference in modeled prospective associations; the study does not establish that CM and DA operate through specific, distinct psychological mechanisms.

The results also clarify the role of personal standards. At the bivariate level, personal standards were weakly associated with anxiety symptoms and choking, and were unrelated to performance. However, after controlling for concern over mistakes and doubts about actions, personal standards predicted lower anxiety symptoms, more flow, and better performance. This pattern is consistent with the argument that the unique component of ambitious standards can be relatively adaptive once the threat-laden and uncertain aspects of perfectionism are separated (Gotwals et al., 2012; Stoeber and Gaudreau, 2017). It should not be interpreted as evidence that raising standards generally reduces anxiety; the sign reversal reflects conditional, mutually adjusted effects.

### 4.2 Concern over mistakes and anxiety symptoms assessed before competition

Concern over mistakes is theoretically relevant to anxiety because errors can acquire broad evaluative meaning for athletes who react strongly to imperfection. Attentional control theory proposes that anxiety can increase worry and stimulus-driven monitoring, reducing the efficiency of goal-directed control (Eysenck et al., 2007), and sport research has linked negative reactions to imperfection with competitive anxiety (Stoeber et al., 2007). The present association between CM and anxiety is consistent with these accounts. While the DASS-21 Anxiety subscale captures a temporally ordered association with general anxiety symptoms, future research using sport-specific measures could further delineate the distinct roles of competitive worry, somatic anxiety, and threat appraisal.

Greater anxiety symptoms were associated with lower flow, greater subjective choking, and poorer performance across all three performance sources. This pattern is broadly compatible with meta-analytic evidence linking cognitive competitive anxiety to poorer sport performance, while recognizing that the DASS-21 construct used here is not equivalent to the sport-specific anxiety measures summarized in that literature (Craft et al., 2003; Woodman and Hardy, 2003). The association with the objective criterion cannot be attributed solely to athletes rating their own performance harshly, but it should be interpreted with caution because the cross-sport objective score was pre-standardized and its source strata cannot be reconstructed from the secondary-analysis archive. The weaker and unstable indirect effect for coach ratings further demonstrates that the three performance sources are not interchangeable (Schweizer et al., 2020).

### 4.3 Direct associations of doubts about actions and candidate mechanisms

Doubts about actions were associated with anxiety symptoms at the bivariate level, but their unique anxiety path was not supported after concern over mistakes was included. This indicates that DA contributed little unique prediction of the DASS-21 anxiety factor in the mutually adjusted model. Nevertheless, DA retained direct associations with all five outcomes. These direct paths are residual structural associations after adjustment for the modeled mediator; they do not clarify why DA were associated with competitive functioning.

Checking, decisional hesitation, reduced trust in prepared responses, conscious reinvestment, perceived preparedness, or movement-specific confidence are plausible candidates for future study, but none were measured here. Explicit-monitoring accounts make some of these possibilities theoretically relevant (Baumeister, 1984; Beilock and Carr, 2001), yet the present data cannot distinguish among them or rule out other explanations. Future studies could combine repeated assessments, process tracing, and direct measures of checking, reinvestment, and decision latency to test whether any of these variables account for the residual associations related to DA.

### 4.4 Measurement implications of flow and choking

Flow and subjective choking were strongly negatively related. This convergence is substantively understandable because effortless, absorbed functioning is unlikely to coexist with the perceived experience of performing below one’s capability under pressure. Nevertheless, latent correlations near −0.91 to −0.95 and HTMT near 0.95 raise a serious discriminant-validity concern. Although the six-factor model fit significantly better than a bipolar model, relative fit superiority does not establish strong empirical distinctiveness. The bipolar model also fit well, and the bifactor analysis assigned most common variance to a general competitive-functioning factor. A cautious interpretation is that the two measures retained some construct-specific variance while largely occupying opposite poles of a common post-competition functioning dimension in this dataset.

The bipolar structural sensitivity model produced the same broad indirect-effect pattern involving CM and DA, indicating that the main statistical comparison was similar when flow and choking were represented by a single functioning factor. Because of their substantial overlap, however, flow and subjective choking should not be treated as independent corroborating outcomes in this dataset. They are better viewed as closely related manifestations of post-competition functioning. Independent validation of the study-specific choking questionnaire, including external criteria and tests across sports and repeated competitions, is needed before stronger claims about its distinct construct validity are warranted.

### 4.5 Theoretical and practical implications

The findings support keeping concern over mistakes and doubts about actions analytically separate when their prospective associations are of interest. Concern over mistakes showed stronger indirect associations through general anxiety symptoms assessed before competition, whereas doubts about actions showed larger residual direct associations. This distinction may help explain heterogeneity in studies that aggregate several perfectionistic concern dimensions into a single score (Kim et al., 2025). At the same time, the present results do not demonstrate a threat mechanism for CM or an action-monitoring mechanism for DA; both remain theoretical interpretations to be tested directly.

Existing intervention evidence provides useful but indirect context. In a randomized trial with varsity women athletes, a brief self-compassion intervention reduced concern over mistakes, as well as self-criticism and rumination (Mosewich et al., 2013). More broadly, a meta-analysis of randomized cognitive-behavioral interventions for perfectionism found reductions in concern over mistakes across predominantly non-sport samples (Galloway et al., 2022), and a trial with Chinese university students reported reductions in both concern over mistakes and doubts about actions following group CBT combined with positive psychotherapy (Zuo and Zhang, 2023). These studies indicate that maladaptive perfectionism dimensions can be modified, but they do not establish that CM and DA require different sport-performance interventions. The present findings therefore provide a rationale for testing dimension-sensitive approaches rather than prescribing specific CM- versus DA-matched strategies.

### 4.6 Strengths, limitations, and future directions

The study has several strengths: a three-wave design centered on a real target competition, item-level latent modeling, simultaneous separation of three perfectionism dimensions, multi-source performance indicators, direct comparison of CM and DA indirect effects, and extensive sensitivity analyses. The 20,000-iteration replication and alternative prior model supported computational stability, while the complete-case and external-performance models alleviated concerns about missing items and common-method dependence. The measurement sensitivity analyses explicitly examined the substantial overlap between flow and subjective choking.

The limitations are equally important. First, temporal ordering does not establish causal mediation. Perfectionism was not randomized, the mediator-outcome relation may be confounded, and baseline anxiety symptoms, flow, choking, and prior performance were not controlled. The estimates are therefore prospective indirect associations rather than confirmed causal effects. Second, the DASS-21 Anxiety subscale is a general symptom measure. Although it was administered 24–48 h before competition, it did not confirm that symptoms were elicited by the competition and cannot distinguish between competition-specific cognitive worry, somatic anxiety, or threat appraisal. Third, the analysis was secondary and not preregistered.

Fourth, missingness was related to concern over mistakes, anxiety symptoms, and years in sport. Available-data Bayesian estimation and complete-case sensitivity reduce, but do not eliminate, bias if missingness depended on unmeasured factors. Fifth, the subjective choking questionnaire was study-specific and has not undergone independent psychometric validation; its acceptable internal consistency and factor loadings are preliminary within-sample evidence only. The flow scale was translated and administered with a five-point response format, and the translated and adapted version also requires further validation. Sixth, the very high overlap between flow and choking means that these outcomes should not be considered fully independent evidence. Seventh, the self- and coach-rated performance indicators were study-specific single items. Eighth, objective performance was harmonized across heterogeneous sport/event/sex groups, which improves broad comparability but precludes the evaluation of sport-specific raw outcomes. Finally, the study did not measure the proposed DA mechanisms, such as reinvestment, checking, or decision latency.

Future work should preregister differentiated pathway models, follow athletes over multiple competitions, and include baseline and repeated measures of anxiety and functioning. Sport-specific anxiety measures could distinguish cognitive worry, somatic arousal, confidence, and facilitative versus debilitative interpretations. Independent samples should validate the choking measure and examine measurement invariance across sport type, sex, and competitive level. Mechanistic studies should directly measure threat appraisal and rumination when testing CM-related hypotheses, as well as checking, reinvestment, hesitation, and action confidence when testing DA-related hypotheses. Intervention trials can then determine whether these dimensions benefit from different forms of performance support.

## 5 Conclusion

Concern over mistakes and doubts about actions were not interchangeable predictors of competitive functioning in the mutually adjusted model. Concern over mistakes showed prospective indirect associations through general anxiety symptoms assessed 24–48 h before competition, resulting in lower flow, greater subjective choking, and poorer self-rated and objective performance. Mistake-related threat was linked to competitive functioning partly through pre-competition anxiety symptoms, whereas doubts about actions were associated mainly through processes not captured by the general anxiety measure. The findings support treating the two evaluative-concern dimensions as related but psychologically distinct.

## Data Availability

The data supporting the conclusions of this article are available from the corresponding author upon reasonable request.

## Glossary

ANX: general anxiety symptoms assessed before competition
AVE: average variance extracted
CFI: Comparative Fit Index
CI: confidence interval
CM: concern over mistakes
DA: doubts about actions
DASS-21: Depression Anxiety Stress Scales-21
ECV: explained common variance
HPD: highest posterior density
HTMT: heterotrait-to-monotrait ratio
PPP: posterior predictive *p*-value
PS: personal standards
PSR: potential scale reduction factor
RMSEA: root mean square error of approximation
SD: standard deviation
SRMR: standardized root mean square residual
STDYX: fully standardized parameterization (Mplus)
TLI: Tucker–Lewis Index
WLSMV: weighted least squares mean- and variance-adjusted (estimator)

## References

[ref1] Baumeister R. F. (1984). Choking under pressure: self-consciousness and paradoxical effects of incentives on skillful performance. J. Pers. Soc. Psychol. 46, 610–620. doi: 10.1037/0022-3514.46.3.610, 6707866

[ref2] Beilock S. L. Carr T. H. (2001). On the fragility of skilled performance: what governs choking under pressure? J. Exp. Psychol. Gen. 130, 701–725. doi: 10.1037/0096-3445.130.4.701, 11757876

[ref3] Craft L. L. Magyar T. M. Becker B. J. Feltz D. L. (2003). The relationship between the competitive state anxiety inventory-2 and sport performance: a meta-analysis. J. Sport Exerc. Psychol. 25, 44–65. doi: 10.1123/jsep.25.1.44

[ref4] Curran T. Hill A. P. (2018). A test of perfectionistic vulnerability following competitive failure among college athletes. J. Sport Exerc. Psychol. 40, 269–279. doi: 10.1123/jsep.2018-0059, 30424701

[ref5] Depaoli S. van de Schoot R. (2017). Improving transparency and replication in Bayesian statistics: the WAMBS-checklist. Psychol. Methods 22, 240–261. doi: 10.1037/met0000065, 26690773

[ref6] Dunn T. J. Baguley T. Brunsden V. (2014). From alpha to omega: a practical solution to the pervasive problem of internal consistency estimation. Br. J. Psychol. 105, 399–412. doi: 10.1111/bjop.12046, 24844115

[ref7] Dunn J. G. H. Causgrove Dunn J. Gotwals J. K. Vallance J. K. H. Craft J. M. Syrotuik D. G. (2006). Establishing construct validity evidence for the sport multidimensional perfectionism scale. Psychol. Sport Exerc. 7, 57–79. doi: 10.1016/j.psychsport.2005.04.003

[ref8] Eysenck M. W. Derakshan N. Santos R. Calvo M. G. (2007). Anxiety and cognitive performance: attentional control theory. Emotion 7, 336–353. doi: 10.1037/1528-3542.7.2.336, 17516812

[ref9] Fornell C. Larcker D. F. (1981). Evaluating structural equation models with unobservable variables and measurement error. J. Mark. Res. 18, 39–50. doi: 10.1177/002224378101800104

[ref10] Frost R. O. Marten P. Lahart C. Rosenblate R. (1990). The dimensions of perfectionism. Cogn. Ther. Res. 14, 449–468. doi: 10.1007/BF01172967

[ref11] Galloway R. Watson H. Greene D. Shafran R. Egan S. J. (2022). The efficacy of randomised controlled trials of cognitive behaviour therapy for perfectionism: a systematic review and meta-analysis. Cogn. Behav. Ther. 51, 170–184. doi: 10.1080/16506073.2021.1952302, 34346282

[ref12] Gaudreau P. Thompson A. (2010). Testing a 2 × 2 model of dispositional perfectionism. Personal. Individ. Differ. 48, 532–537. doi: 10.1016/j.paid.2009.11.031

[ref13] Gotwals J. K. Dunn J. G. H. (2009). A multi-method multi-analytic approach to establishing internal construct validity evidence: the sport multidimensional perfectionism scale 2. Meas. Phys. Educ. Exerc. Sci. 13, 71–92. doi: 10.1080/10913670902812663

[ref14] Gotwals J. K. Stoeber J. Dunn J. G. H. Stoll O. (2012). Are perfectionistic strivings in sport adaptive? A systematic review of confirmatory, contradictory, and mixed evidence. Can. Psychol. 53, 263–279. doi: 10.1037/a0030288

[ref15] Harris D. J. Allen K. L. Vine S. J. Wilson M. R. (2023). A systematic review and meta-analysis of the relationship between flow states and performance. Int. Rev. Sport Exerc. Psychol. 16, 693–721. doi: 10.1080/1750984X.2021.1929402

[ref16] Henry J. D. Crawford J. R. (2005). The short-form version of the depression anxiety stress scales (DASS-21): construct validity and normative data in a large non-clinical sample. Br. J. Clin. Psychol. 44, 227–239. doi: 10.1348/014466505X29657, 16004657

[ref17] Henseler J. Ringle C. M. Sarstedt M. (2015). A new criterion for assessing discriminant validity in variance-based structural equation modeling. J. Acad. Mark. Sci. 43, 115–135. doi: 10.1007/s11747-014-0403-8

[ref18] Hewitt P. L. Flett G. L. (1991). Perfectionism in the self and social contexts: conceptualization, assessment, and association with psychopathology. J. Pers. Soc. Psychol. 60, 456–470. doi: 10.1037/0022-3514.60.3.456, 2027080

[ref19] Hill A. P. Curran T. (2016). Multidimensional perfectionism and burnout: a meta-analysis. Personal. Soc. Psychol. Rev. 20, 269–288. doi: 10.1177/1088868315596286, 26231736

[ref20] Hill A. P. Hanton S. Matthews N. Fleming S. (2010). Choking in sport: a review. Int. Rev. Sport Exerc. Psychol. 3, 24–39. doi: 10.1080/17509840903301199

[ref21] Hill A. P. Mallinson-Howard S. H. Jowett G. E. (2018). Multidimensional perfectionism in sport: a meta-analytical review. Sport Exerc. Perform. Psychol. 7, 235–270. doi: 10.1037/spy0000125

[ref22] Hill A. P. Mallinson-Howard S. H. Madigan D. J. Jowett G. E. (2020). “Perfectionism in sport, dance, and exercise: an extended review and reanalysis,” in Handbook of Sport Psychology, eds. Tenenbaum G. Eklund R. C.. 4th ed (Hoboken, NJ: Wiley), 121–157.

[ref23] Jackson S. A. Eklund R. C. (2002). Assessing flow in physical activity: the flow state scale-2 and dispositional flow scale-2. J. Sport Exerc. Psychol. 24, 133–150. doi: 10.1123/jsep.24.2.133

[ref24] Jones G. (1995). More than just a game: research developments and issues in competitive anxiety in sport. Br. J. Psychol. 86, 449–478. doi: 10.1111/j.2044-8295.1995.tb02565.x, 8542197

[ref25] Kim H. Madigan D. J. Hill A. P. (2025). Multidimensional perfectionism and sport performance: a systematic review and meta-analysis. Int. Rev. Sport Exerc. Psychol. 1–34. doi: 10.1080/1750984X.2025.2541342

[ref26] Lizmore M. R. Dunn J. G. H. Causgrove Dunn J. Hill A. P. (2019). Perfectionism and performance following failure in a competitive golf-putting task. Psychol. Sport Exerc. 45:101582. doi: 10.1016/j.psychsport.2019.101582

[ref27] Lovibond P. F. Lovibond S. H. (1995a). The structure of negative emotional states: comparison of the depression anxiety stress scales (DASS) with the Beck depression and anxiety inventories. Behav. Res. Ther. 33, 335–343. doi: 10.1016/0005-7967(94)00075-U, 7726811

[ref28] Lovibond S. H. Lovibond P. F. (1995b). Manual for the Depression Anxiety Stress Scales. 2nd Edn Washington, DC: Psychology Foundation.

[ref29] Maxwell S. E. Cole D. A. (2007). Bias in cross-sectional analyses of longitudinal mediation. Psychol. Methods 12, 23–44. doi: 10.1037/1082-989X.12.1.23, 17402810

[ref30] Miočević M. O’Rourke H. P. MacKinnon D. P. Brown H. C. (2018). Statistical properties of four effect-size measures for mediation models. Behav. Res. Methods 50, 285–301. doi: 10.3758/s13428-017-0870-1, 28342072 PMC5809552

[ref31] Mosewich A. D. Crocker P. R. E. Kowalski K. C. DeLongis A. (2013). Applying self-compassion in sport: an intervention with women athletes. J. Sport Exerc. Psychol. 35, 514–524. doi: 10.1123/jsep.35.5.514, 24197719

[ref32] Muthén B. Asparouhov T. (2012). Bayesian structural equation modeling: a more flexible representation of substantive theory. Psychol. Methods 17, 313–335. doi: 10.1037/a0026802, 22962886

[ref33] Muthén L. K. Muthén B. O. (2017). Mplus User’s Guide. 8th Edn Los Angeles, CA: Muthén & Muthén.

[ref34] Nordin-Bates S. M. Madigan D. J. Hill A. P. Olsson L. F. (2024). Perfectionism and performance in sport: exploring non-linear relationships with track and field athletes. Psychol. Sport Exerc. 70:102552. doi: 10.1016/j.psychsport.2023.102552, 37913912

[ref35] Norsworthy C. Dimmock J. A. Miller D. J. Krause A. Jackson B. (2023). Psychological flow scale (PFS): development and preliminary validation of a new flow instrument that measures the core experience of flow to reflect recent conceptual advancements. Int. J. Appl. Posit. Psychol. 8, 309–337. doi: 10.1007/s41042-023-00092-8

[ref36] Rodriguez A. Reise S. P. Haviland M. G. (2016). Evaluating bifactor models: calculating and interpreting statistical indices. Psychol. Methods 21, 137–150. doi: 10.1037/met0000045, 26523435

[ref37] Schweizer G. Furley P. Rost N. Barth K. (2020). Reliable measurement in sport psychology: the case of performance outcome measures. Psychol. Sport Exerc. 48:101663. doi: 10.1016/j.psychsport.2020.101663

[ref38] Stoeber J. (2011). The dual nature of perfectionism in sports: relationships with emotion, motivation, and performance. Int. Rev. Sport Exerc. Psychol. 4, 128–145. doi: 10.1080/1750984X.2011.604789

[ref39] Stoeber J. Gaudreau P. (2017). The advantages of partialling perfectionistic strivings and perfectionistic concerns: critical issues and recommendations. Personal. Individ. Differ. 104, 379–386. doi: 10.1016/j.paid.2016.08.039

[ref40] Stoeber J. Otto K. (2006). Positive conceptions of perfectionism: approaches, evidence, challenges. Personal. Soc. Psychol. Rev. 10, 295–319. doi: 10.1207/s15327957pspr1004_2, 17201590

[ref41] Stoeber J. Otto K. Pescheck E. Becker C. Stoll O. (2007). Perfectionism and competitive anxiety in athletes: differentiating striving for perfection and negative reactions to imperfection. Personal. Individ. Differ. 42, 959–969. doi: 10.1016/j.paid.2006.09.006

[ref42] Stoeber J. Uphill M. A. Hotham S. (2009). Predicting race performance in triathlon: the role of perfectionism, achievement goals, and personal goal setting. J. Sport Exerc. Psychol. 31, 211–245. doi: 10.1123/jsep.31.2.211, 19454772

[ref43] Swann C. Keegan R. J. Piggott D. Crust L. (2012). A systematic review of the experience, occurrence, and controllability of flow states in elite sport. Psychol. Sport Exerc. 13, 807–819. doi: 10.1016/j.psychsport.2012.05.006

[ref44] Woodman T. Hardy L. (2003). The relative impact of cognitive anxiety and self-confidence upon sport performance: a meta-analysis. J. Sports Sci. 21, 443–457. doi: 10.1080/0264041031000101809, 12846532

[ref45] Zuo Z. Zhang X. (2023). A randomized controlled trial of group CBT with positive psychotherapy intervention for university students with maladaptive perfectionism in China. Front. Psychol. 14:1161575. doi: 10.3389/fpsyg.2023.1161575, 37539006 PMC10395325

[ref46] Zyphur M. J. Oswald F. L. (2015). Bayesian estimation and inference: a user’s guide. J. Manag. 41, 390–420. doi: 10.1177/0149206313501200

